# Supplementary material for: Identifying substitutional oxygen as a prolific point defect in monolayer transition metal dichalcogenides
Source: Nat Commun. 2019 Jul 29;10:3382. doi: 10.1038/s41467-019-11342-2 (PMC6662818; doi:10.1038/s41467-019-11342-2)
Supplement: Supplementary file 1 — Supplementary Information [file 41467_2019_11342_MOESM1_ESM.pdf]

Supplementary Information for

Identifying substitutional oxygen as a prolific point defect in monolayer transition metal dichalcogenides

S. Barja, *et al*.

Supplementary Figure 1 shows a typical STM image on 2D-WS_2 growth by chemical vapor deposition (CVD) on multilayer graphene (MLG) on SiC. We observed two different types of individual point defects with six-fold symmetry: a daisy-like shape (left defect in Supplementary Figure 1(b) and (c)); and donut-like shape (right defect in Supplementary Figure 1(b) and (d)). Supported by nc-AFM images acquired with a CO-functionalized tip we identify two O substituted sulfur atom (**O**$_S$): (1) The one atom size depression (left defect in Supplementary Figure 1(e) and (f)) indicates an apparently missing S atom in the upper S-sublattice facing the tip; and (2) the apparently increased height of a S atom (right defect in Supplementary Figure 1(e) and (g)) can be attributed to a protruding S atom in the top S layer in response to a **O**$_S$ defect in the bottom S-sublattice facing the underlying graphene layer. The described relaxation agrees with DFT geometry relaxation of the defect structure, similar to the **O**$_{Se}$ defect in $MoSe_2$ shown in Fig. 3(a)-(c) in the main manuscript.

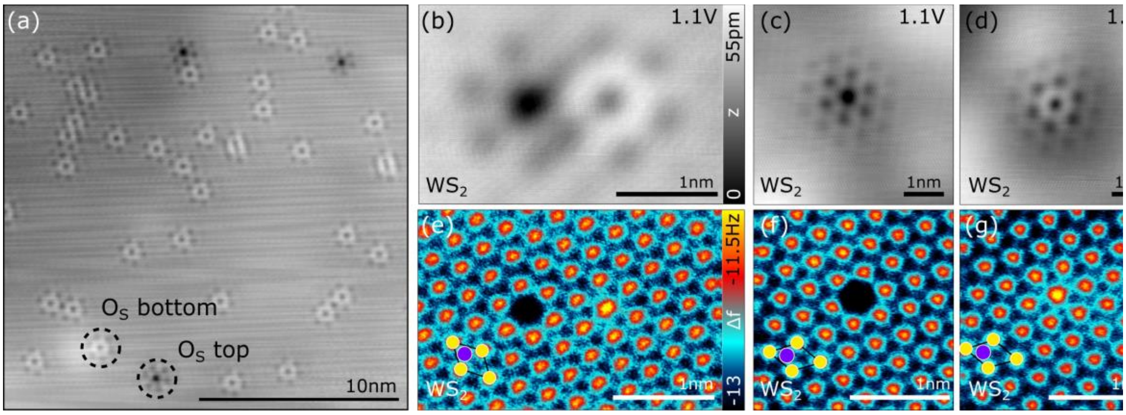

Supplementary Figure 1. (a) Large scale STM image of a WS_2 region showing a high density of point defects. The two different types of defect symmetry can be identified as single O-substituted S atoms placed either in the top (**O**$_S$ top) or in the bottom (**O**$_S$ bottom) S-sublattices. (b) Close-up view STM image at the conduction band of an **O**$_S$ top (left defect) and O_S bottom (right defect) next to each other. Notice that the STM contrast around each defect remains unaltered compared to the contrast observed for the isolated **O**$_S$ top (c) and **O**$_S$ bottom (d) defects. (e), (f) and (g) show the nc-AFM images measured on the same area and with the same tip as the STM images in (b), (c) and (d), respectively.

Atomic-scale characterization of top and bottom substitutional oxygen in $MoSe_2$

The 2D-$MoSe_2$ sample was grown by molecular beam epitaxy (MBE) on bilayer graphene (BLG) on SiC. In order to protect the $MoSe_2$ monolayer from contamination or adsorbates during the transport in air to the UHV-STM chamber, we deposited a Se capping layer with a thickness of about 10 nm, which subsequently was removed by annealing the sample at about 600K in the UHV-STM and then transfer to the STM for surface characterization.

We explore the most abundant type of point defects on $MoSe_2$ and identify substitutional oxygen in a Se atom site ($\mathbf{O}_{Se}$) as argued in the paper and outlined in detail below. Figure 1 in the main manuscript shows the nc-AFM image of two different types of such $\mathbf{O}_{Se}$ defects: (1) The one atom size depression in Fig. 1 (a) on the left is a $\mathbf{O}_{Se}$ in the upper Se-sublattice facing the tip (denoted $\mathbf{O}_{Se}$ top); and (2) the apparently increased height of a Se atom on the right could be identified as a $\mathbf{O}_{Se}$ in the bottom Se-sublattice facing the underlying graphene layer tip (denoted $\mathbf{O}_{Se}$ bottom), slightly lifting the above laying Se atom facing the tip. We want to stress that the identification of the $\mathbf{O}_{Se}$ top and bottom defect, and in general any type of defect in 2D-TMDs, is extremely challenging. We can only solve the defect structure by comparing nc-AFM measurements to nc-AFM calculations and scanning tunneling spectroscopy (STS) to advanced *ab initio* electronic structure calculations. Neither of those methods could reveal the structure assignment alone. Importantly, *ab initio* calculations were essential to establish a sound foundation for the structure assignment. Our structure assignment is based on the entirety of the following observations: (i) Both the top and bottom defect version exhibit an identical electronic defect signature as measured by STS (see Supplementary Figure 2 (a)). This is a strong indication that both defects belong to the same type of defect. (ii) The nc-AFM image reveals that the defect is located on a Se site with an apparently missing Se atom on the top surface layer in one case and a protruding Se atom in the other one (See Fig. 1). (iii) The simulated nc-AFM image of a Se vacancy and a substitutional defects considering H and O would, in principle, fit the observed nc-AFM contrast (see Fig. 3). This narrows down considerably the most probable defect possibilities. (iv) STS does not observe any evidence of deep in-gap defect states (see Fig. 2), which is expected for both a Se vacancy

and the Substitutional H defect (see Supplementary Figure 5). Several options, including defect charging (see Supplementary Figure 3 and Supplementary Figure 5), or canceling of tunneling matrix elements due to symmetry reasons, that could prevent us from detecting such an in-gap state can be ruled out. We considered the case that the wavefunctions composing the tip- and defect-related states might be orthogonal, leading to a vanishing tunneling matrix element and vanishing current related to these states. However, this scenario is unlikely based on the experimental evidence at hand: the defect was probed with metallic and CO-functionalized tips, which have been shown to exhibit states having s- and p-wave character. Neither of these two orbital symmetries detected an in-gap state. (v) The spatial distribution of the calculated local density of states of $\mathbf{O}_{Se}$ at the conduction and valence bad edge resemble the experimental dI/dV images at the corresponding voltages (see Fig. 4). (vi) We found an analogous behavior to all points discussed above for substitutional oxygen in WS_2 grown by chemical vapor deposition (Fig. 2, Supplementary Figure 1), which anticipates a general trend for semiconducting TMDs in general.

Note that similar STM features have been previously discussed as vacancies or substitutional atoms of different kinds in 2D-TMDs, indicating the challenging structure assignment of TMD defects and the current inconsistency in the community and the understanding of they real influence on their electronic properties of the materials[1–7].

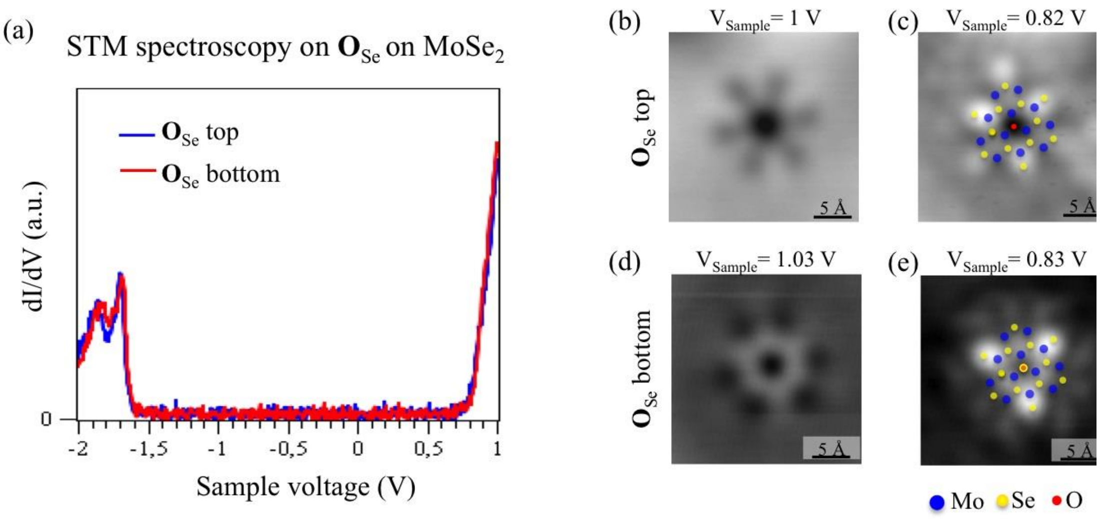

Supplementary Figure 2. (a) STM dI/dV spectrum acquired on an O substituent Se defect (**O**$_{Se}$) on $MoSe_2$, placed on the bottom Se-sublattice (red) shows the exact same spectroscopic features than the dI/dV spectrum acquire on the **O**$_{Se}$ placed on the top Se-sublattice (blue). The dI/dV spectra were measured with the same tip and in the same sample area. STM images from the (b) top **O**$_{Se}$ and (d) bottom **O**$_{Se}$ at voltages about 200mV above the conduction band, respectively, show a six-fold symmetry, instead of the three-fold symmetry characteristic from the STM images on the (c) top **O**$_{Se}$ and (e) bottom **O**$_{Se}$ at the conduction band edge.

Challenging the presence of an in-gap defect state

As outlined in the manuscript, there are several known mechanisms that can preclude detecting a localized in-gap state to be detected by STS, particularly due to dynamic or static charging of the defect. In Supplementary Figure 3 (a) we compare STS spectra taken at a typical tunneling set point of I = 3nA at V = 1V with spectra taken at about 3Å closer. Apparently, there are no additional defect resonances observed but additional peaks associated with direct tunneling into the underlying graphene. This suggests that tunneling from a deep in-gap state to the graphene should be readily established since even a tip that is several Ångstroms farther away is able to directly tunnel into graphene with a considerable tunneling current of several nA at voltages as low as 10mV. This is true unless the charged defect is stabilized by atomic relaxations in the film. Atomic relaxations on ionic films have been shown to stabilize different charge states of individual atoms[8]. Different charge states can be discriminated[9] and charge state switching[10] detected by Kelvin probe spectroscopy. Supplementary Figure 3 (b) shows the Kelvin probe

parabolas measured on the $\mathbf{O}_{Se}$ (blue) and on the bare substrate (green). In the bias range probed no charge state switching was observed. In addition, the local contact potential difference corresponding to the bias at the vertex point indicates that the defect is neutral.

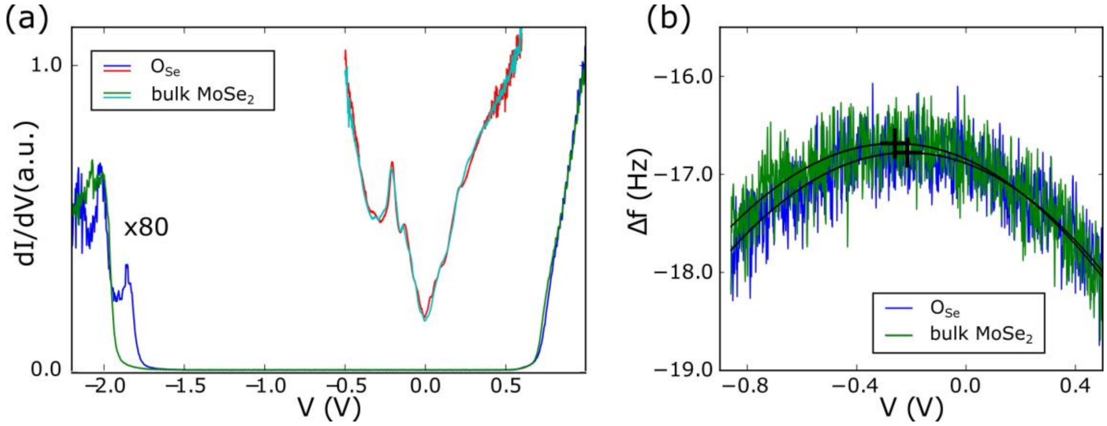

Supplementary Figure 3. (a) dI/dV spectra recorded on the $\mathbf{O}_{Se}$ top (blue, red) and on the bare substrate (green, cyan). The spectra were taken at different tip heights: at a set point of I = 3nA at V = 1V (blue, green) and I = 600nA at V = 0.6V (red, cyan), respectively. (b) Kelvin probe parabolas recorded on the $\mathbf{O}_{Se}$ top (blue) and on the bare substrate (green).

To further test this scenario, we compare the energy-dependent spatial distribution of the DOS around the defect we observe in the experiment with the simulated ones for the case of both, the bare Se vacancy and the O substitutional defect. Figure 4 shows the experimental dI/dV constant-height conductance maps, compared to simulated images using DFT wavefunction slices of $\mathbf{V}_{Se}$ and $\mathbf{O}_{Se}$ valence and conduction band edges. The charge distributions show a three-fold symmetry, extending spatially over two (pristine) lattice constants from the defect. In Supplementary Figure 4 (c) we show the spatial distribution of the DOS from the theoretically predicted in-gap state for the pristine $\mathbf{V}_{Se}$, which exhibits a similar symmetry, but does not fit the experimentally observe registry to both Se- and Mo-sublattice at the defect conduction band, excluding the possibility of an energy shift due to charging of the in-gap state into the defect conduction band.

Finally, the fact that both the top and bottom chalcogen vacancies are electronically equivalent (Supplementary Figure 2 (a)) represents a strong indicative of the negligible influence of the graphene substrate and defect-substrate interactions.

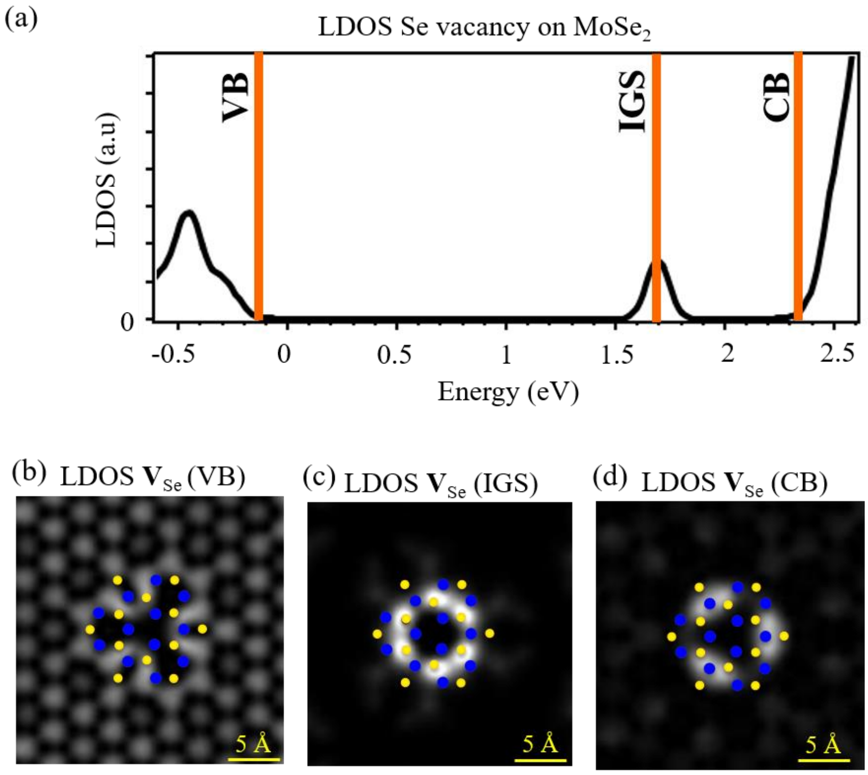

Supplementary Figure 4. (a) Calculated LDOS of monolayer $MoSe_2$ with Se vacancies within the GW approach. The Fermi level (0 eV) is placed at the valence band onset. We observe a gap between non-defect states of 1.9 eV and the presence of a doubly-degenerated unoccupied in gap state, ~0.4 eV below the non-defect conduction band minimum. (b) Simulated LDOS from DFT wavefunction slice of the bare $\mathbf{V}_{Se}$ valence band (VB), (c) the in-gap state (IGS) and (d) the conduction band (CB) edge. Calculated LDOS are compatible with the three-fold symmetry and spatial extent observed in the experimental images, at the defect site. However, the simulated LDOS of the predicted defect in-gap state does not resemble any shape we observed experimentally.

DFT calculations on a various point-defects in monolayer $MoSe_2$ and their effect on the density of states

To study the effect of point defects on the presence of localized in-gap state in monolayer $MoSe_2$, we perform Density functional theory (DFT) calculations. All DFT calculations were performed within the Quantum-ESPRESSO[11] package using the PBE exchange-correlation functional[12] and with norm-conserving pseudopotentials[11,13,14]. Plane wave basis set with a kinetic energy cutoff of 80 Ry was used for the wavefunctions. The atomic structures of supercells of up to 7x7 times the pristine unit-cell, containing the point defects were relaxed self-consistently with a 15x15 k-point mesh, and a monolayer-monolayer separation of at least 15 Å of vacuum in the out-of-plane direction. For the calculation of monolayer $MoSe_2$ on graphene, a slight tensile strain in the graphene layer (with a lattice constant of 2.5 Å) was imposed in order to match the 5x5 supercell of $MoSe_2$. The dispersion correction presented in Ref.15[15] was included in this case in order to describe interaction between graphene and $MoSe_2$.

Supplementary Figure 5 shows the resulting DFT density of states for the different configurations: a pristine monolayer as reference (black), a monolayer containing neutral Se vacancies (red), a monolayer containing hydrogenated Se vacancies, with hydrogen atom bound to the vacancy site (blue), a monolayer containing negatively charged Se vacancies (green) with an additional electron, and a monolayer containing neutral Se vacancy in a monolayer $MoSe_2$ deposited on graphene (orange). For all examined defects, DFT results suggest the appearance of defect-localized in-gap states, as discussed in the main text.

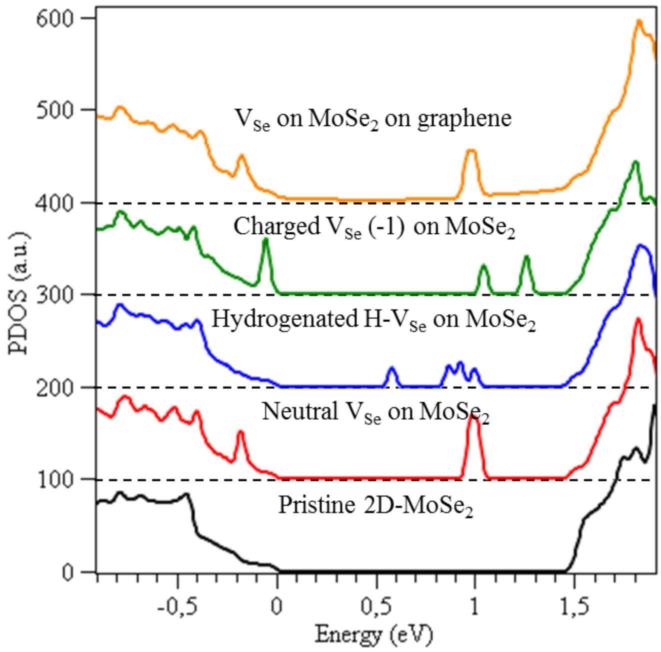

Supplementary Figure 5. Calculated DFT density of states (DOS) of pristine single-layer $MoSe_2$ (black line); and defective system containing one Se vacancy neutral (red line), hydrogenated (blue line), charged with one electron (green), and with the monolayer of $MoSe_2$ deposited on graphene (orange). All the spectra have been aligned with the conduction band edge at 0 eV.

GW calculations of a Se vacancy and O substituent point defects in monolayer $MoSe_2$

We first performed DFT calculations within the local density approximation (LDA)[16] using the Quantum-ESPRESSO code[11]. The calculations were done on a 5x5 supercell arrangement of the $MoSe_2$ monolayer with either one Se vacancy or with a single O atom instead of one of the Se atoms. We used a plane-wave basis and norm-conserving pseudopotentials with a 70 Ry plane-wave cutoff. We included the Mo semi-core 4d, 4p and 4s states as valence states for our DFT and GW calculations. The distance between repeated supercells in the out-of-plane direction was 15 Å. We fully relaxed the geometry and included spin-orbit interactions as a perturbation. We performed the GW calculation within the BerkeleyGW code[17] using the generalized plasmon-pole model[18] and an energy cutoff of 25 Ry for the screening function. For the Se vacancy, we used 1677 unoccupied states, and tested the convergence with respect to unoccupied states by comparing to a calculation using 50,000 effective states obtained by averaging high-energy states

within a small energy window. We verified convergence with respect to the number of bands, as discussed in Ref. 19[19]. For the O substituent defect, we used 1245 and 1673 empty bands, and interpolated to the limit of infinite bands. For this defect we also verified the energy gap convergence at a higher screening cutoff of 35 Ry. In order to speed the convergence with respect to k-point sampling, we employed non-uniform sampling of the Brillouin zone, where the smallest q-vector corresponds to ∼1/1150th of a reciprocal lattice vector[20].

Supplementary References

1. Liu, X., Balla, I., Bergeron, H. & Hersam, M. C. Point Defects and Grain Boundaries in Rotationally Commensurate MoS_2 on Epitaxial Graphene. *J. Phys. Chem. C* **120,** 20798–20805 (2016).
2. Vancsó, P. *et al.* The intrinsic defect structure of exfoliated MoS_2 single layers revealed by Scanning Tunneling Microscopy. *Sci. Rep.* **6,** 29726 (2016).
3. Peng, J.-P. *et al.* Molecular beam epitaxy growth and scanning tunneling microscopy study of $TiSe_2$ ultrathin films. *Phys. Rev. B* **91,** 121113 (2015).
4. Hildebrand, B. *et al.* Doping Nature of Native Defects in 1T-$TiSe_2$. *Phys. Rev. Lett.* **112,** 197001 (2014).
5. Zhang, S. *et al.* Defect Structure of Localized Excitons in a WSe_2 Monolayer. *Phys. Rev. Lett.* **119,** 046101 (2017).
6. Li, H. *et al.* Activating and optimizing $MoSe_2$ basal planes for hydrogen evolution through the formation of strained sulphur vacancies. *Nat. Mater.* **15,** 48–53 (2016).
7. Pető, J. *et al.* Spontaneous doping of the basal plane of MoS_2 single layers through oxygen substitution under ambient conditions. *Nat. Chem.* **10,** 1246–1251 (2018).
8. Repp, J., Meyer, G., Olsson, F. E. & Persson, M. Controlling the Charge State of Individual Gold Adatoms. *Science* **305,** 493–495 (2004).
9. Gross, L. *et al.* Measuring the Charge State of an Adatom with Noncontact Atomic Force Microscopy. *Science* **324,** 1428–1431 (2009).
10. Steurer, W., Fatayer, S., Gross, L. & Meyer, G. Probe-based measurement of lateral single-electron transfer between individual molecules. *Nat. Commun.* **6,** 8353 (2015).
11. Giannozzi, P. *et al.* QUANTUM ESPRESSO: a modular and open-source software project for quantum simulations of materials. *J. Phys. Condens. Matter* **21,** 395502 (2009).
12. Perdew, J. J. P. J., Burke, K. & Ernzerhof, M. Generalized Gradient Approximation Made Simple. *Phys. Rev. Lett.* **77,** 3865–3868 (1996).
13. Vanderbilt, D. Soft self-consistent pseudopotentials in a generalized eigenvalue formalism. *Phys. Rev. B* **41,** 7892–7895 (1990).
14. Lejaeghere, K. *et al.* Reproducibility in density functional theory calculations of solids. *Science* **351,** aad3000-aad3000 (2016).
15. Grimme, S. Semiempirical GGA-type density functional constructed with a long-range dispersion correction. *J. Comput. Chem.* **27,** 1787–1799 (2006).
16. Kohn, W. & Sham, L. J. Self-Consistent Equations Including Exchange and Correlation Effects. *Phys. Rev.* **140,** A1133–A1138 (1965).
17. Deslippe, J. *et al.* BerkeleyGW: A massively parallel computer package for the calculation of the quasiparticle and optical properties of materials and nanostructures. *Comput. Phys. Commun.* **183,** 1269–1289 (2012).
18. Hybertsen, M. S. & Louie, S. G. Electron correlation in semiconductors and insulators: Band gaps and quasiparticle energies. *Phys. Rev. B* **34,** 5390–5413 (1986).
19. Refaely-Abramson, S., Qiu, D. Y., Louie, S. G. & Neaton, J. B. Defect-induced modification of low-lying excitons and valley selectivity in monolayer transition metal dichalcogenides. *Phys. Rev. Lett.* **121,** 167402 (2018).
20. da Jornada, F. H., Qiu, D. Y. & Louie, S. G. Nonuniform sampling schemes of the Brillouin zone for many-electron perturbation-theory calculations in reduced dimensionality. *Phys. Rev. B* **95,** 035109 (2017).
